# A Novel Robotic Technique for Mapping Patellofemoral Kinematics in Total Knee Arthroplasty

**DOI:** 10.1016/j.artd.2024.101610

**Published:** 2025-01-24

**Authors:** Edward O’Bryan, Christopher Jones, Samuel Joseph

**Affiliations:** a Linacre Private Hospital (Ramsay Health), Hampton, Victoria, Australia; b Department of Orthopaedics, Peninsula Health, Frankston, Victoria, Australia; c Department of Orthopaedics, Alfred Health, Melbourne, Victoria, Australia

**Keywords:** Robotic, Computer-assisted, Patellofemoral, Knee, Arthroplasty, Patella

## Abstract

Computer assistance has significantly improved precision in total knee arthroplasty (TKA). Current robotic systems address tibiofemoral kinematics, ignoring the patellofemoral joint. This described TKA technique allows assessment and adjustment of patellofemoral kinematics. Reproducible landmarks on the tibial tubercle, trochlea, and patella are defined. With the “Special Point” function of the CORI surgical system, arcs of points are recorded dynamically. This records trochlear groove translation, patellar tracking, patellar tilt, and tibial tubercle rotation. During implant trials, special points are recollected to determine how these four parameters have changed compared to the native knee. Component adjustments can be made to optimize patellofemoral kinematics without compromising tibiofemoral balance. This may be a tool to mitigate patellofemoral maltracking and may improve TKA outcomes. Further studies are required to investigate outcomes.

## Introduction

The introduction of computer-assisted surgery (CAS) has increased the reliability and accuracy of measuring, planning, and cutting bony surfaces in total knee arthroplasty (TKA) [1]. Optical tracking systems offer error margins of less than 1 mm in translation, and less than 1° in angulation [1]. Studies have thus demonstrated improved precision in executing the planned tri-planar cuts of both the femur and tibia [2,3]. As optical tracking systems refresh rates continually improve, dynamic measurements in space become increasingly feasible and reliable [4,5]. This facilitates the implementation of more complex alignment philosophies in TKA. An example is “functional alignment”, in which the native joint line obliquity and constitutional alignment are respected, and components are aligned within this framework to optimize the compartment gaps in the coronal plane [[6], [7], [8]]. Default software on currently available robotic knee systems generally restricts assessment to alignment and dynamic motion of the tibiofemoral articulation; patellofemoral kinematics are largely ignored. With continued improvements in the accuracy of robotic technology, it follows that greater precision of implantation is expected, such as addressing the patellofemoral articulation.

Patellofemoral problems such as maltracking or anterior knee pain following TKA remain a common source of revision [9,10], and revision surgery to address these problems is not always successful [11]. These problems are also implicated as a source of dissatisfaction following TKA [10,12,13]. This article describes a novel technique to assess and adjust patellofemoral kinematics during TKA using a currently available robotic surgical system (CORI; Smith & Nephew), giving the surgeon the ability to assess and optimize patellofemoral alignment and kinematics in real time during the index procedure.

The described technique uses the “Special Point” function of the CORI surgical system. The “Special Point” function allows the surgeon to use an optically tracked probe to record a series of points anywhere in space during bone mapping, irrespective of whether that point is in contact with bone or not. This feature can map points on both the patella and the tibia in relation to the femoral array, thereby defining the position of the patella and tibia relative to the femur. These points can be mapped prior to any bone cuts to define native positions, and can be mapped again after resurfacing to detect any changes in patellar or tibial positions relative to the femoral array. Component adjustments can then be made where relevant to optimize patellofemoral mechanics. This assessment of the dynamic relationships of the patellofemoral and tibiofemoral joints has not otherwise been described using robotic systems currently available.

## Surgical technique

The patient and robotic system (including femoral and tibial arrays) are positioned as per the surgeon’s preferred set-up. For this technique, it is important for the limb to be reproducibly positioned at 90° as well as 30°, allowing assessment of the tibial station and alignment of the patella as it engages the trochlea [14].

Reproducible landmarks on the tibial tubercle and the patella are required. We insert the tibial checkpoint into the center of the tibial tubercle for this purpose; an electrocautery mark can be used as an alternative. The precise location of the tubercle landmark is not critical, because changes in the position of the tubercle relative to the femur are being assessed rather than the precise location of the tubercle itself. The patellar landmark is located on the subcutaneous surface of the patella, opposite the articular point of contact of the patella with the trochlea. This location helps to prevent unnatural patellar tilt occurring during mapping secondary to pressure from the probe. Although a checkpoint can be used for this landmark, we prefer an electrocautery mark to prevent a stress point which could predispose the patella to fracture [12]. Once the surgeon has appropriately mapped the femur, the femur special point collection function is used.

**Figure 1:**
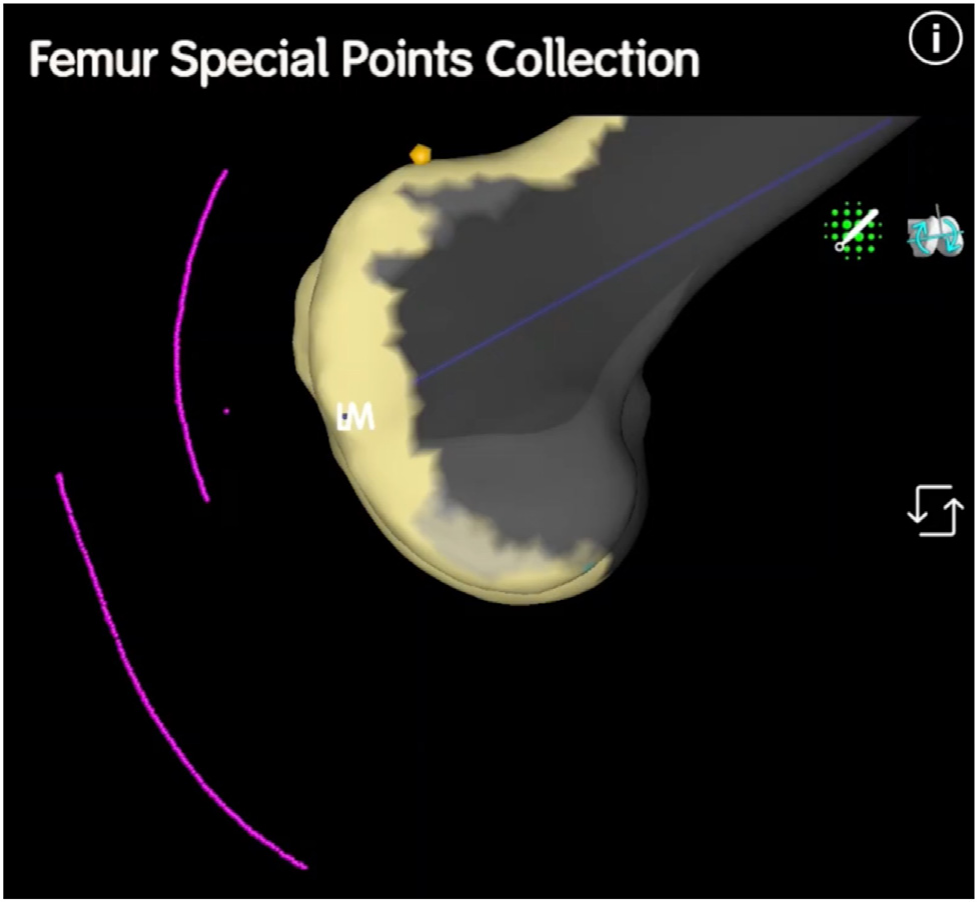
A lateral view of the distal femur. Special points (in purple) denote the sagittal plane path of the subcutaneous border of the patella and the tibial tubercle.

The knee is positioned at 90° of flexion with the patella everted. Whiteside’s line is mapped using an arc of special points within the deepest path of the trochlear groove, defining the trochlear offset and trajectory of the patellofemoral joint (Fig. 1).

The patella is reduced to its native position, and a special point is made at the predefined tibial landmark (ie, the tibial checkpoint) with the knee at 90° of flexion. This defines a spatial position of the tibial tubercle in relation to the femur, which defines the sagittal tibial offset and tibial rotation in this position.

**Figure 2:**
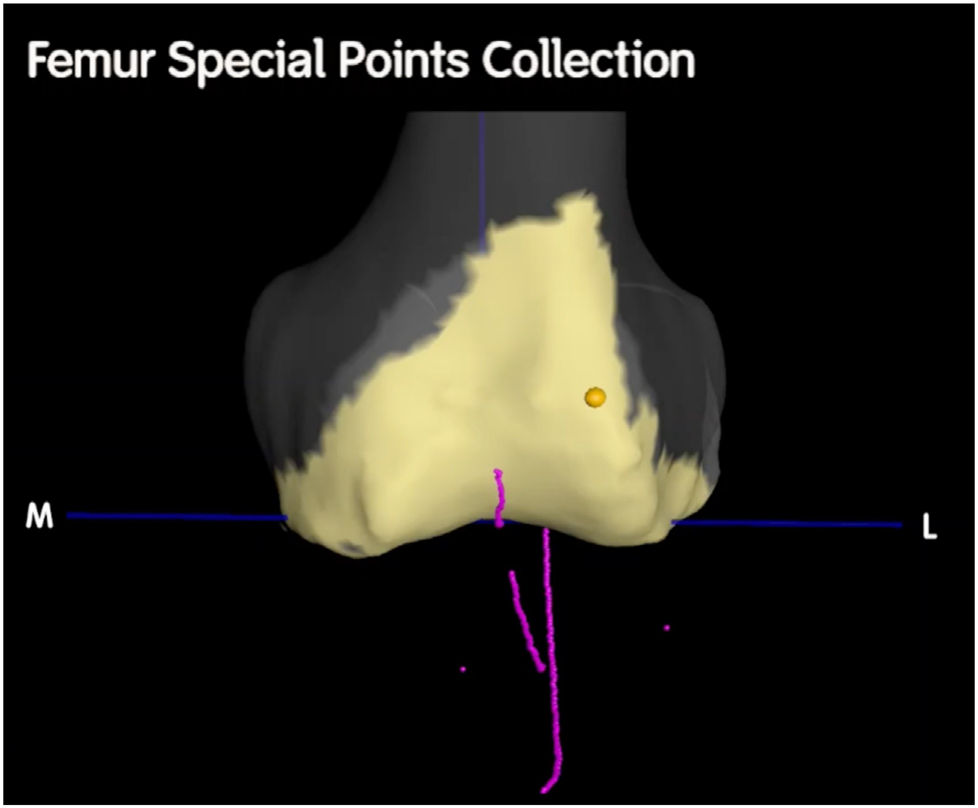
A frontal view of the distal femur in extension. Special points denote the trochlear groove, the tibial tubercle, and patella tracking (2 singular points were recorded on medial and lateral aspects of the patella for this case in 90° of flexion to denote preoperative tilt).

With the probe firmly seated in the tibial checkpoint, the knee is gently extended while continuing to record special points. This creates an arc demonstrating the position of the tibial tubercle in relation to the femur from 90° of flexion to full extension. A kinematic tibiofemoral axis and tibial tubercle-trochlear groove distance (TT-TG) is thus established (Figure 1, Figure 2).

The knee is then positioned at 30° of flexion. With the probe seated on the patellar point, taking care not to artificially induce abnormal patellar translation or tilt (which could be highly user dependent), the knee is gently flexed to 90° while recording special points (Figure 1, Figure 2). This creates an arc that defines patellar tracking. To assess patellar tilt, medial and lateral patella points can be mapped throughout the 30°-90° flexion range. A minimum of 3 noncollinear points are required to map the patella in 6 degrees of freedom [15].

The surgeon then continues the standard CORI software workflow; to map the tibia, assess the gap balance, plan the component positioning in-line with their desired philosophy, and resurface the femur and tibia.

**Figure 3:**
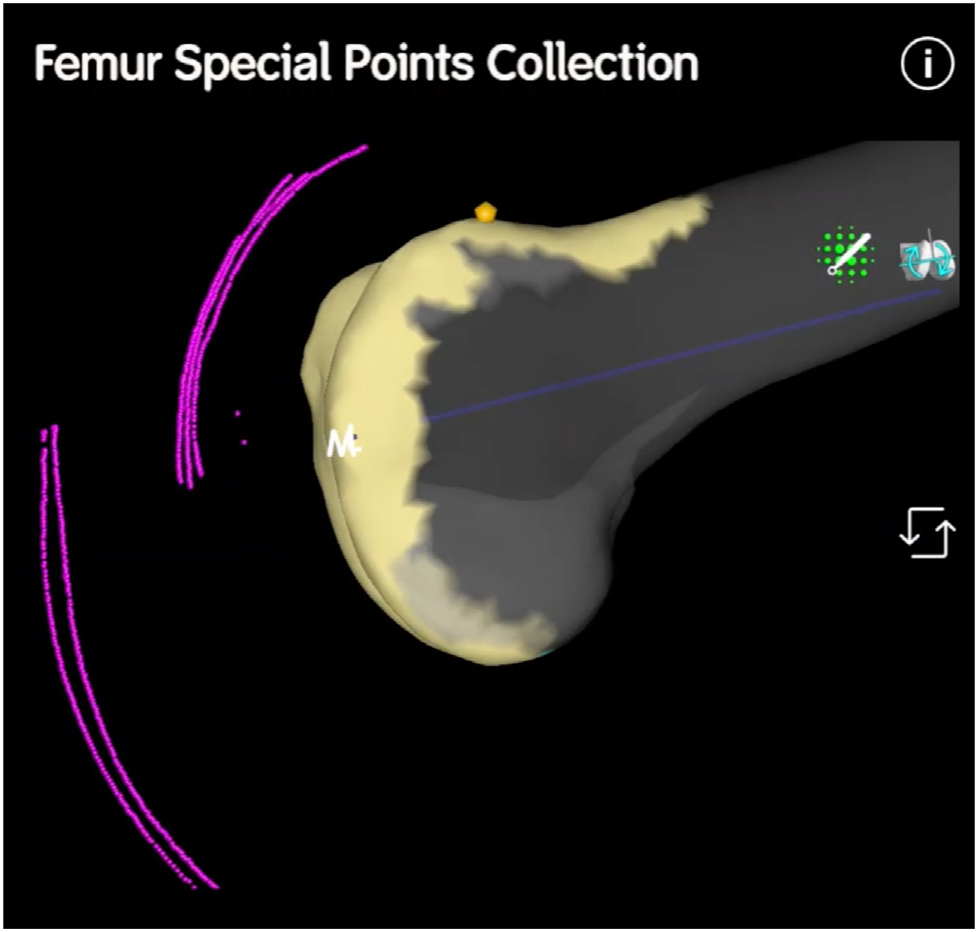
A lateral view of the distal femur during the trial stage. The tibial sagittal offset is slightly increased in this case, accounting for cartilage wear that has been restored. Three lines denote patellar tracking from the preoperative, trial, and resurfaced stages.

During the trial stage, prior to patellar resurfacing, the special points are then recollected (Fig. 3). The touch-screen function can be used to manipulate the view of the distal femur and special points, allowing a frontal, oblique, or lateral view relative to the distal femur.

The lateral view allows assessment of the patellar height and the sagittal offset of the trochlear groove, patella, and tibial tubercle (Fig. 3). The frontal view allows assessment of the trochlear groove translation, patellar tracking, patellar tilt, and tibial tubercle rotation.

At this point, the surgeon can make component adjustments to optimize tracking. This may include translation or rotation of the tibial tray to restore the tibial tubercle, translation of the femoral component to restore the trochlear groove, or adjusting the polyethylene thickness to restore sagittal tibial offset [16]. The special points can be rechecked to confirm restoration of the native anatomy.

Patellar offset needs to be considered in the context of trochlear offset. Trochlear offset is typically reduced due to femoral component size and positioning [17,18], and patellar offset will be equally affected. Resurfacing of the patella can then be performed with an appropriate resection depth to restore the extensor mechanism to its native position after accounting for preoperative patellofemoral wear. Unless tibial or femoral component alignment requires further adjustment, abnormality in patellar tilt can be addressed at this stage by adjusting the translation of the patella button, the plane of patella resection, or soft tissue release with the goal to restore the subcutaneous patellar surface to its native position [19]. Restoring the offset and plane of the subcutaneous patella allows restoration of the extensor lever arm, irrespective of changes to the trochlear groove, which may have been necessary to balance the tibiofemoral articulation.

## Case example

**Figure 4:**
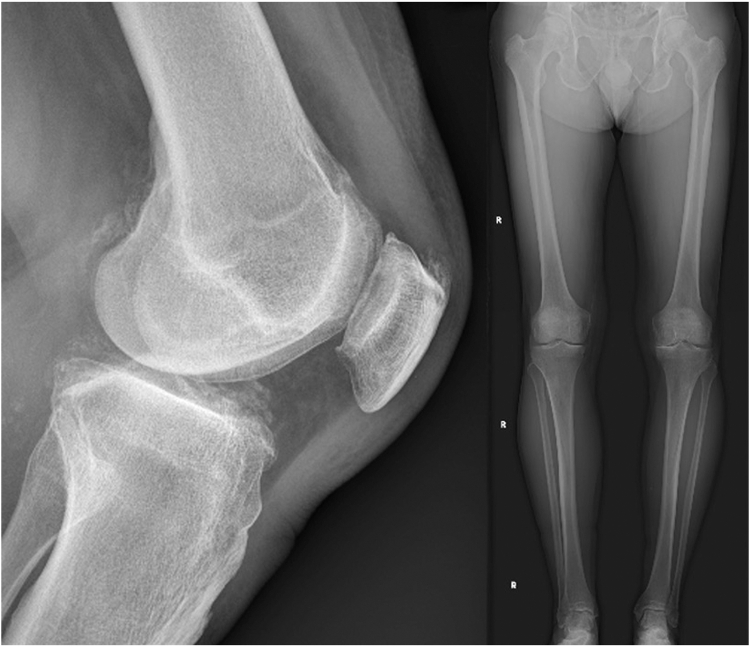
Preoperative imaging demonstrating unicompartmental disease and 6° of varus due to cartilage loss. The patient was CPAK type 1 with 4° of constitutional varus. The patella was positioned at a normal height. CPAK, coronal plane alignment of the knee.

**Figure 5:**
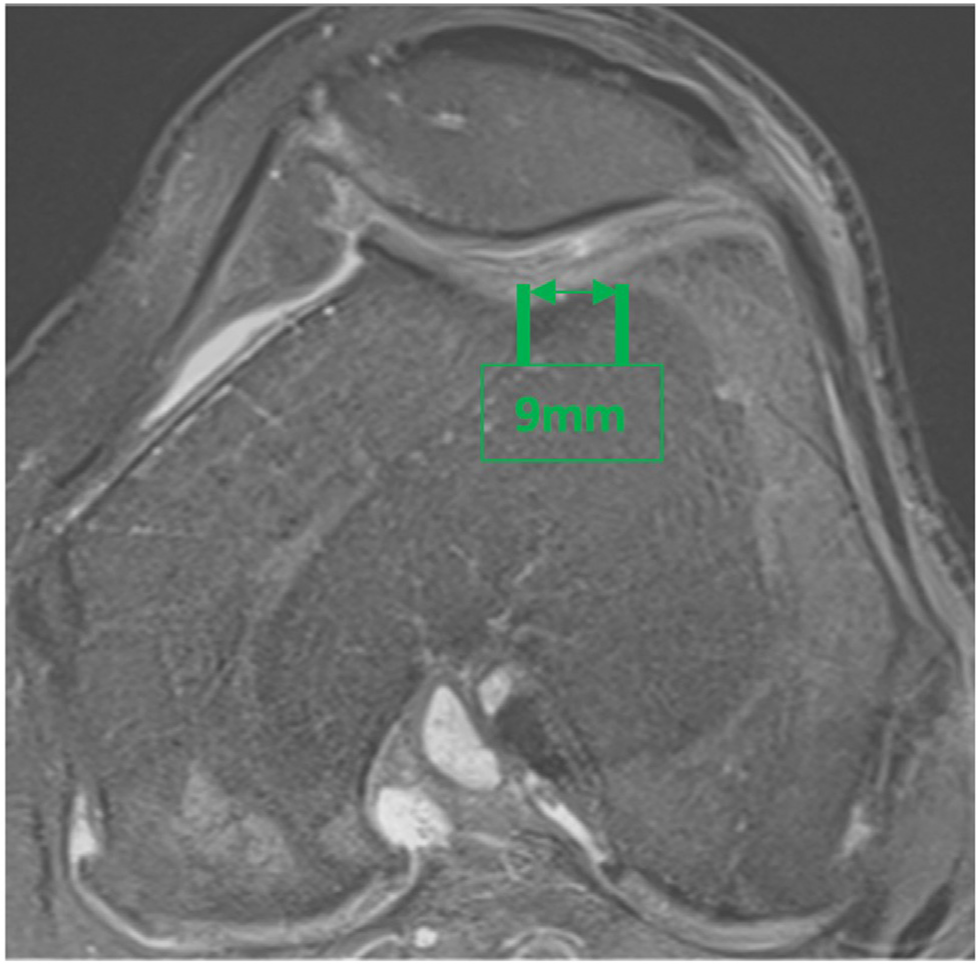
A preoperative MRI demonstrated intact patellofemoral cartilage. The tibial tubercle-trochlear groove distance was 9 mm. MRI, magnetic resonance imaging.

The following case describes a total knee replacement performed for a 74-year-old male with isolated medial compartment osteoarthritis and normal patella tracking. The patient was coronal plane alignment of the knee type 1, with 4° of constitutional varus and a Caton-Deschamps ratio of 0.9 (Fig. 4). The TT-TG was 9 mm (Fig. 5).

**Figure 6:**
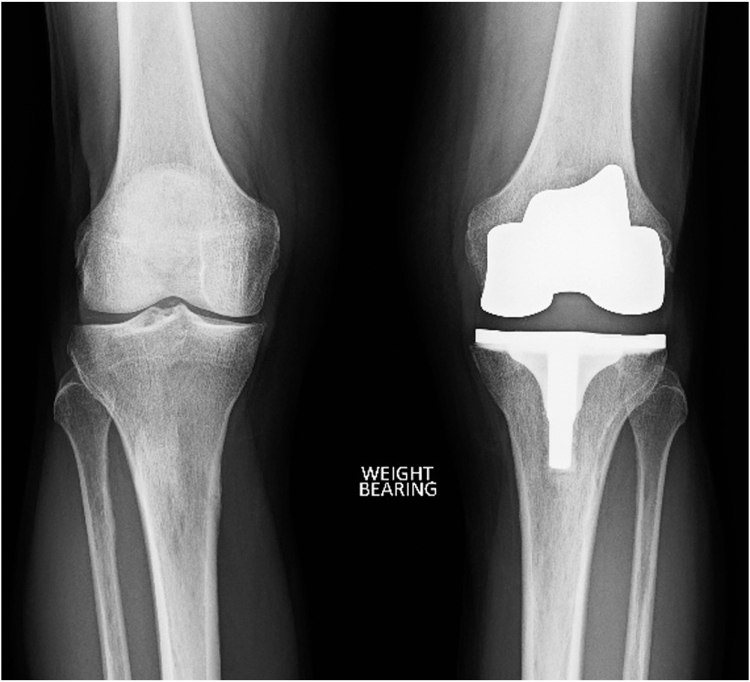
The patient’s constitutional alignment was restored postoperatively. The implant is a Journey 2 BCS (Smith & Nephew), with an asymmetric polyethylene design.

Given the preoperative patellofemoral state was normal with no cartilage loss, the surgical plan was to restore the patella to this position after resurfacing. The femur was resurfaced with matched resections, and only minor adjustments to the component positions to balance the coronal gaps (Fig. 6).

**Figure 7:**
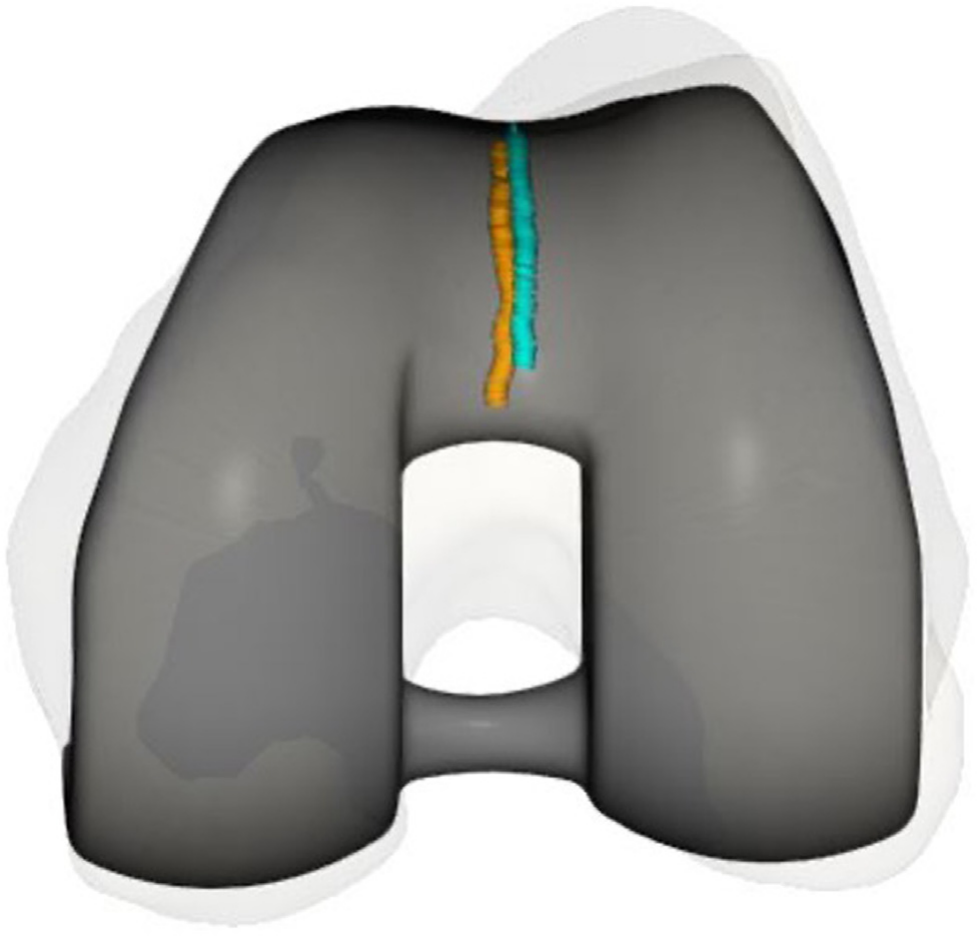
Trochlear groove restoration. The preresurfaced groove is depicted in aqua and the trial implant groove is depicted in orange. The trajectory and offset of the groove is restored, and is subtly medialized by up to 1 mm.

**Figure 8:**
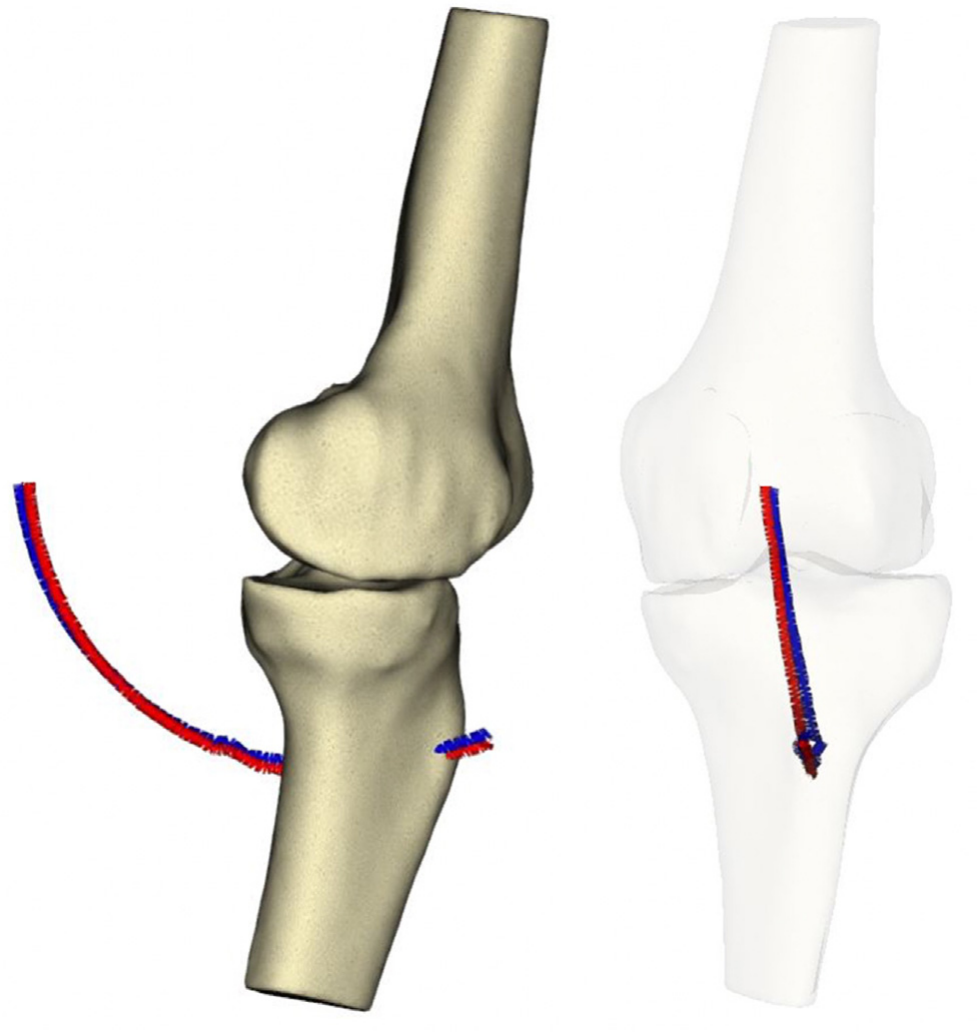
The tibiofemoral kinematic axis from extension to 90°. The blue line depicts the preresurfaced tibial tubercle and the red line depicts the trial stage tibial tubercle. The resurfaced TT-TG was subtly decreased by 1 mm, and the sagittal offset was restored. TT-TG, tibial tubercle-trochlear groove distance.

**Figure 9:**
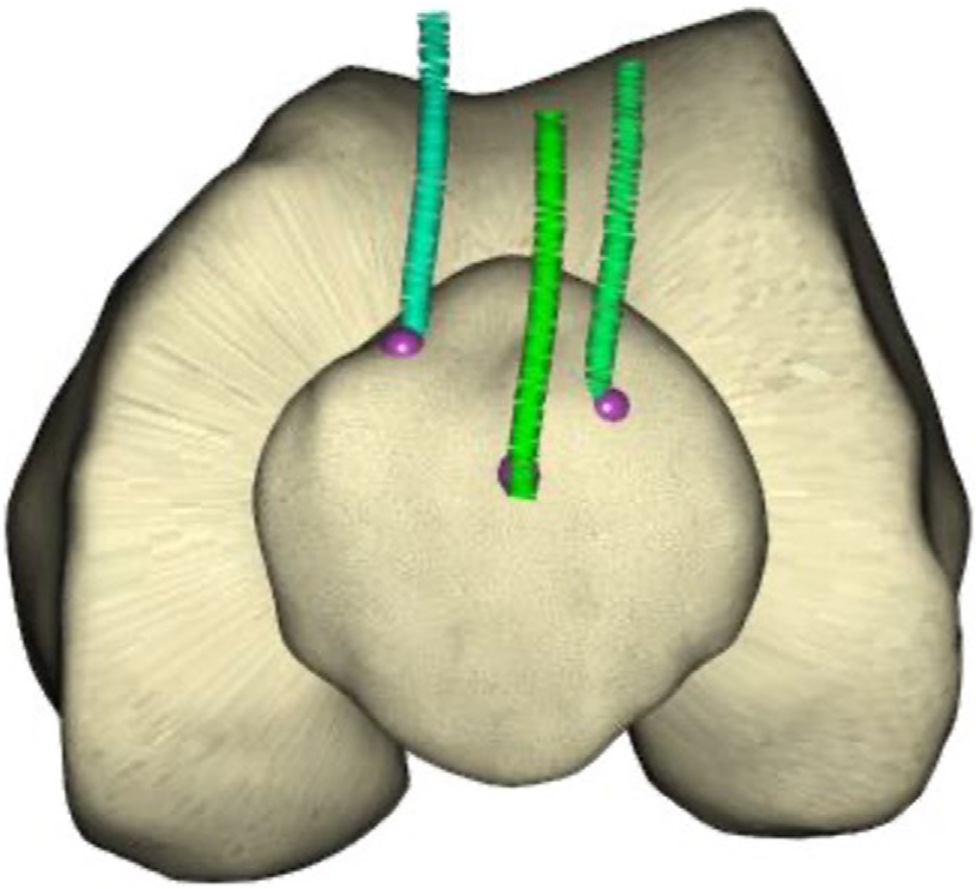
A representation of 3 noncolinear points on the patella through a range of motion. The precise position of the points upon the patella is a demonstration only.

**Figure 10:**
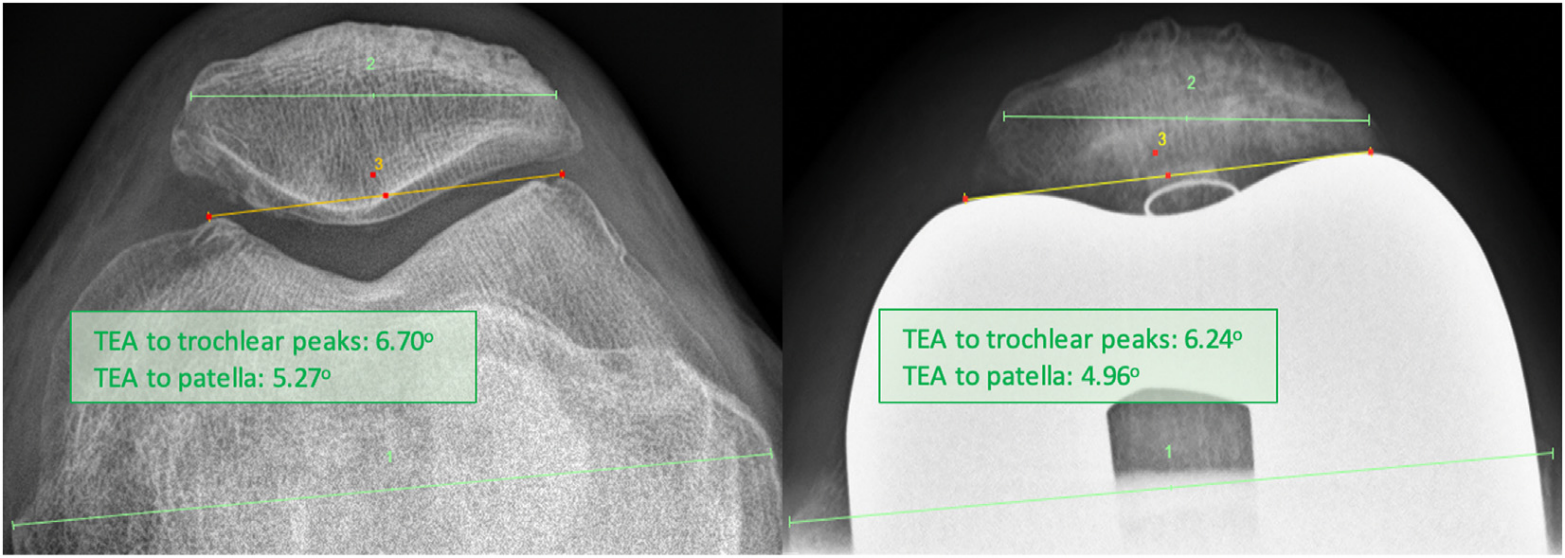
Preoperative and postoperative Skyline views. The trans-epicondylar axis (TEA) has been used as a reference plane to compare the plane intersecting medial and lateral native peaks, as well as the sclerotic margin underlying the subcutaneous border of the patella. The subcutaneous border of the patella was restored to its native position within half of a degree.

The femoral component was positioned to restore the trochlear groove offset and trajectory (Fig. 7). The tibial tray was aligned to optimize the kinematic axis, sagittal offset, and TT-TG restoration between the preresurfaced and trial stages (Fig. 8). Figure 9 demonstrates 3 noncolinear points measured on the patella through a range of motion, which were restored postoperatively. Skyline views compare the preoperative and postoperative patella position (Fig. 10) [20].

## Discussion

While the use of CAS has steadily increased for tibiofemoral resurfacing, and while robotic systems are available for trochlea resurfacing, there is currently no system available that offers dynamic assessment of patellar kinematics [9,21,22]. As the use of robotics has increased, alignment philosophies that deviate from the mechanical axis to accurately restore coronal balance have followed. Restoring the native patellofemoral joint kinematics can also be achieved with CAS techniques.

This technique uses the CORI special point function to objectively measure the native patellofemoral joint kinematics, albeit in the diseased knee. By comparing these points in the resurfaced knee, the surgeon can determine which aspect of the extensor mechanism requires adjustment in the event of an overstuffed joint or maltracking.

Conventional techniques have been described to measure and restore the sagittal tibial offset [16]. This requires a caliper measurement from the distal femur and is therefore prone to human error in creating a reproducible measurement. The conventional technique also assumes that the distal femur is anatomically restored, as a raised joint-line will reduce offset without a difference in caliper measurement. This technique can assess sagittal tibial offset relative to the femoral shaft (the optical tracker), independent of the distal femoral cut.

It is uncommon for any of the described parameters to deviate meaningfully from their native position after a well-balanced, functionally aligned knee. In most cases, this technique is therefore an objective confirmation that patellofemoral kinematics are restored, where this is otherwise difficult to measure with any precision or objectivity in TKA. However, the rate of patellofemoral maltracking is reported in the literature to be an issue in 1%-20% of TKA [13]. If the patellar offset or patellar tracking is abnormal during the trial phase, this technique efficiently aids the surgeon in identifying what aspects of the complex patellofemoral and tibiofemoral articulation are likely to be causative, and therefore help to correct the problem without unnecessary soft-tissue releases.

While cartilage loss has not been mentioned in the technique description, this is an important factor for native joint restoration. It is common to observe cartilage loss of both the patella and trochlea, which leads to a combined reduction in patellofemoral offset, or abnormal patella tilt during the mapping phase. Equally, cartilage loss of the distal femoral and tibial surfaces results in reduced sagittal tibial offset compared to the prediseased knee. It is therefore pertinent that the surgeon accounts for cartilage loss, insofar that measuring the patellofemoral or tibiofemoral kinematics are intended to restore the knee to a prediseased soft-tissue balance. Therefore, the desired position of the resurfaced knee may deviate from the special points that are registered in the diseased state in any given TKA.

## Summary

This article describes a novel technique using the special point function of the CORI robotic system to map the femoral, tibial, and patellar aspects of patellofemoral tracking. Restoration of these parameters can be observed using the robot during the trial phase, and fine adjustments to component positioning can be guided by the technique to help restore native patellofemoral kinematics.

## Conflicts of interest

Christopher Jones has given paid presentations for Smith & Nephew. Samuel Joseph has given paid presentations for Smith & Nephew. All other authors declare no potential conflicts of interest.

## CRediT authorship contribution statement

**Edward O’Bryan:** Writing – review & editing, Writing – original draft, Methodology, Formal analysis, Conceptualization. **Christopher Jones:** Writing – review & editing, Supervision, Project administration, Conceptualization. **Samuel Joseph:** Writing – review & editing, Supervision.

## Appendix A Supplementary data

Conflict of Interest Statement for Jones

Conflict of Interest Statement for O’Bryan

Conflict of Interest Statement for Joseph
